# Crystal structure of 2-oxo-2*H*-chromen-3-yl 4-chloro­benzoate and Hirshfeld surface analysis

**DOI:** 10.1107/S2056989016019538

**Published:** 2017-01-01

**Authors:** Eric Ziki, Siaka Sosso, Frédérica Mansilla-Koblavi, Abdoulaye Djandé, Rita Kakou-Yao

**Affiliations:** a Laboratoire de Cristallographie et Physique Moléculaire, UFR SSMT, Université Félix Houphouët Boigny de Cocody 22 BP 582 Abidjan 22, Côte d’Ivoire; b Laboratoire de Chimie Moléculaire et Matériaux, Equipe de Chimie Organique et Phytochimie, Université Ouaga I Pr Joseph KI-ZERBO 03 BP 7021 Ouagadougou 03, Burkina Faso

**Keywords:** crystal structure, chromane, hydrogen bond, π–π inter­actions, quantum-chemical calculations, Hirshfeld surface analysis

## Abstract

In the title compound, C_16_H_9_ClO_4_ the dihedral angle between the coumarin ring system [maximum deviation = 0.023 (1) Å] and the benzene ring is 73.95 (8)°. In the crystal, π–π inter­actions link the dimers into a three-dimensional framework. A quantum chemical calculation is in generally good agreement with the observed structure, although the calculated dihedral angle between the ring systems (85.7%) is somewhat larger than the observed value [73.95 (8)°]. Hirshfeld surface analysis has been used to confirm and qu­antify the supra­molecular inter­actions.

## Abstract 2

In the title compound, C_16_H_9_ClO_4_, the dihedral angle between the coumarin ring system [maximum deviation = 0.023 (1) Å] and the benzene ring is 73.95 (8)°.

## Chemical context   

Coumarin and its derivatives are widely recognized for their multiple biological activities, including anti­cancer (Lacy *et al.*, 2004 ▸; Kostova, 2005 ▸), anti-inflammatory (Todeschini *et al.*, 1998 ▸), anti­viral (Borges *et al.*, 2005 ▸), anti-malarial (Agarwal *et al.*, 2005 ▸) and anti­coagulant (Maurer *et al.*, 1998 ▸) properties. As part of our studies in this area, we now describe the synthesis and crystal structure of the title compound, (I).
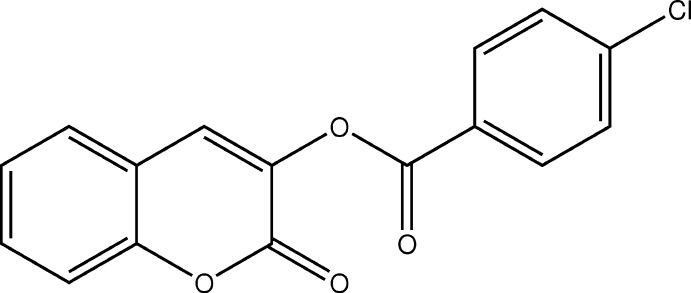


## Structural commentary   

**Figure 1:**
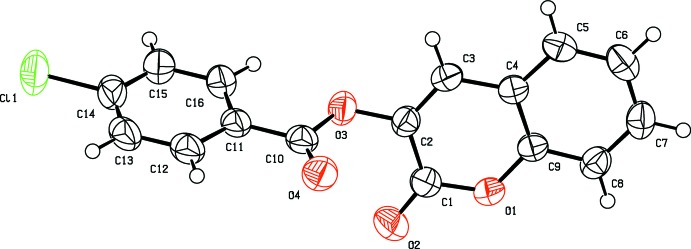
The mol­ecular structure of compound (I), with displacement ellipsoids drawn at the 50% probability level.

In compound (I) (Fig. 1 ▸), the coumarin ring system is, as expected, almost planar [maximum deviation = 0.023 (1) Å] and is oriented at an angle of 73.95 (8)° with respect to the benzene ring. An inspection of the bond lengths shows that there is a slight asymmetry of the electronic distribution around the coumarin ring: the C3—C2 [1.335 (2) Å] and C2—C1 [1.456 (2) Å] bond lengths are shorter and longer, respectively, than those excepted for a C_ar_—C_ar_ bond. This suggests that the electronic density is preferentially located in the C2—C3 bond at the pyrone ring, as seen in other coumarin derivatives (Gomes *et al.*, 2016 ▸; Ziki *et al.*, 2016 ▸).

## Supra­molecular features   

**Figure 2:**
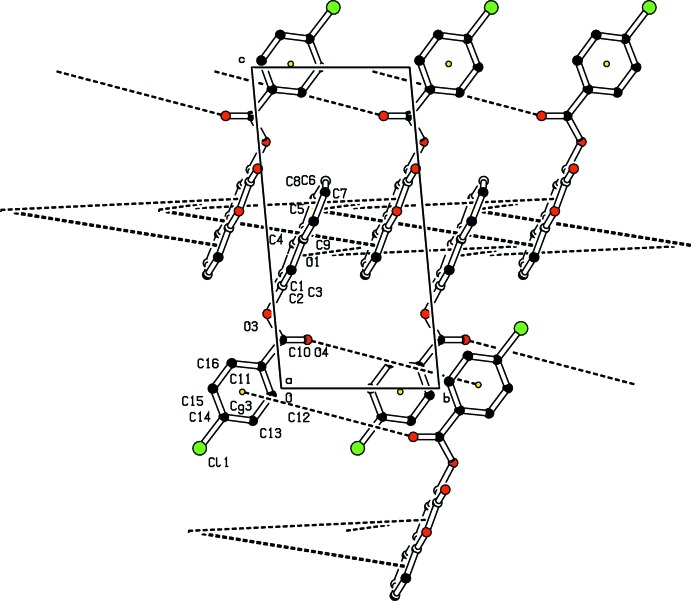
Partial packing diagram for (I), showing the π–π stacking and C—O⋯π inter­actions (dashed lines). The yellow dots are ring centroids. H atoms have been omitted for clarity.

In the crystal, weak aromatic π–π stacking inter­actions (Janiak, 2000 ▸) are present [*Cg*1⋯*Cg*2(1 − *x*, −*y*, 1 − *z*) = 3.4781 (10) Å and *Cg*2⋯*Cg*2(1 − *x*, 1 − *y*,1 − *z*) = 3.5644 (11) Å, where *Cg*1 is the centroid of the coumarin pyran ring and *Cg*2 is the centroid of the coumarin benzene ring], thus forming a three-dimensional supra­molecular network. A weak C11=O4⋯*Cg*3(1 − *x*, − *y*, −*z*) (π-ring) inter­action between O4 and a symmetry-related benzene ring (C6–C11, centroid *Cg*3) of is also present (Fig. 2 ▸).

## Hirshfeld surface analysis   

**Figure 3:**
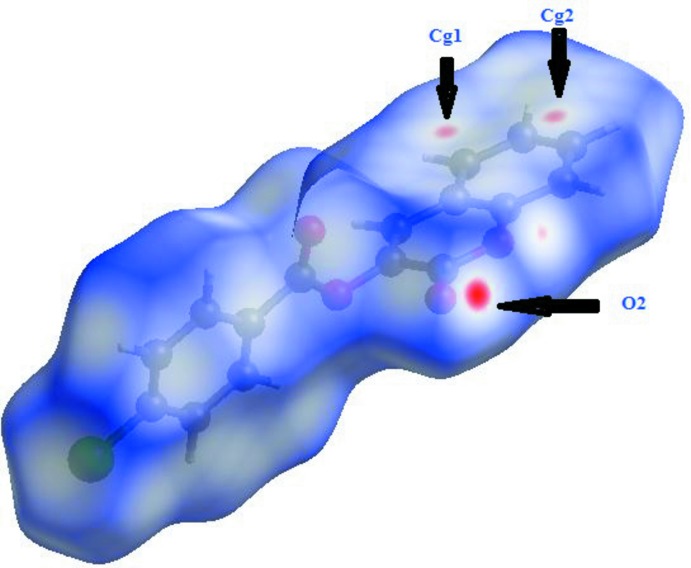
A view of the Hirshfeld surface mapped over *d*_norm_. The contact points (red) are labelled to indicate the atoms participating in the inter­molecular inter­actions.

**Figure 4:**
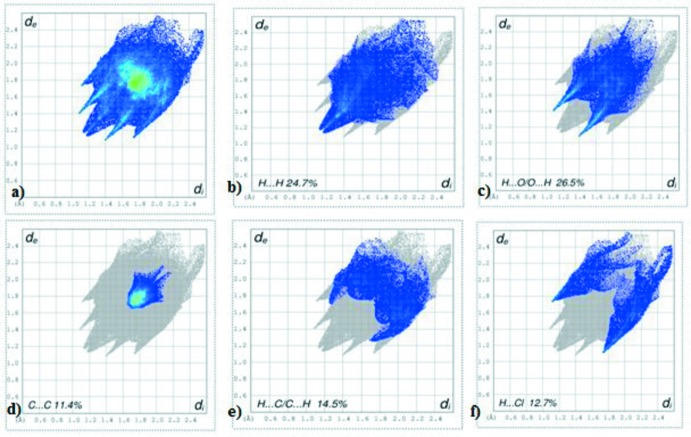
Two-dimensional fingerprint plots: (*a*) overall, and delineated into contributions from different contacts: (*b*) H⋯H, (*c*) H⋯O/O⋯H, (*d*) C⋯C, (*e*) H⋯C/C⋯H and (*f*) H⋯Cl/Cl⋯H.

*Crystal Explorer3.1* (Wolff *et al.*, 2012 ▸) was used to generate the Hirshfeld surface and two-dimensional fingerprint (FP) plots (Rohl *et al.*, 2008 ▸). The analysis of intra­molecular and inter­molecular inter­actions through the mapping of *d*_norm_ is permitted by the contact distances *d*_i_ and *d*_e_ from the Hirshfeld surface to the nearest atom inside and outside, respectively. In compound (I), there are four O atoms and a Cl atom that can potentially act as acceptors for hydrogen bonds, but one of O atoms and the H atom of the chloro­benzoate moiety are involved in the establishment of intra­molecular hydrogen bonds. The surface mapped over *d*_norm_ displays four red spots that correspond to areas of close contact between the surface and the neighbouring environment and is shown in Fig. 3 ▸. The contributions from different contacts were selected by partial analysis of the FP plots (Fig. 4 ▸). C⋯C contacts correspond to inter­molecular π–π inter­actions.

The greatest contribution (26.5%) is from the H⋯O/O⋯H contacts, which appear as the highlighted red spot on the side of the surface (Figs. 3 ▸ and 4*c* ▸). The red spots in the middle of the surface correspond to C⋯C contacts appearing near *d*_e_ = *d*_i_ ≃1.7 and 1.8 Å (Fig. 4*d* ▸). As expected in organic compounds, the H⋯H contacts are important with a 24.7% contribution to Hirshfeld surface (Fig. 4*b* ▸). There are also H⋯C/C⋯H and H⋯Cl/Cl⋯H contacts, which make contributions of 14.5 and 12.7%, respectively (Figs. 4*e* and 4*f* ▸).

## Quantum-chemical calculations   

Quantum-chemical calculations were performed and the results compared with the experimental analysis. An *ab-initio* Hartree–Fock (HF) method was used with the standard 6-31G basis set using the *GAUSSIAN03* software package (Frisch *et al.*, 2004 ▸; Dennington *et al.*, 2007 ▸) to obtain the optimized mol­ecular structure. The computational results are in good agreement with the experimental crystallographic data (see Supplementary Tables S1 and S2). The dihedral angle between the coumarin ring and the chloro­benzoate ring for the calculated structure is 85.7°, which is larger than the value of 73.95 (8)° for the observed structure.

## Synthesis and crystallization   

To a solution of 4-chloro­benzoyl chloride (6.17 × 10 ^−3^ mol ≃ 0.8 ml) in dry tetra­hydro­furan (31 ml) was introduced dried tri­ethyl­amine (3 molar equivalents ≃ 2.6 ml). While stirring strongly, 6.17 × 10 ^−3^ mol (1 g) of chroman-2,3-dione was added in small portions over 30 min. The reaction mixture was then refluxed for 4 h and poured into a separating funnel containing 40 ml of chloro­form. The solution was acidified with dilute hydro­chloro­ric acid until the pH was 2–3. The organic layer was extracted, washed with water until neutral, dried over MgSO_4_ and the solvent removed. The resulting precipitate (crude product) was filtered off with suction, washed with petroleum ether and dissolved in a minimum of di­chloro­methane by heating under agitation. Hexane was added to this hot mixture until the formation of a new precipitate started, which dissolved in the resulting mixture upon heating. Upon cooling, yellow crystals of the title compound precipitated in a yield of 70%; m.p. 478–482 K.

## Refinement details   

Crystal data, data collection and structure refinement details are summarized in Table 1 ▸. H atoms were placed in calculated positions (C—H = 0.93 Å) and refined using a riding-model approximation with *U*_iso_(H) = 1.2*U*_eq_(C).

## Supplementary Material

Crystal structure: contains datablock(s) I. DOI: 10.1107/S2056989016019538/hb7629sup1.cif

Structure factors: contains datablock(s) I. DOI: 10.1107/S2056989016019538/hb7629Isup2.hkl

Supporting information file. DOI: 10.1107/S2056989016019538/hb7629Isup3.cml

CCDC reference: 1521043

Additional supporting information: crystallographic information; 3D view; checkCIF report

## Figures and Tables

**Table 1:** Experimental details

Crystal data	
Chemical formula	C_16_H_9_ClO_4_
*M*_r_	300.68
Crystal system, space group	Triclinic, *P* 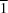
Temperature (K)	293
*a*, *b*, *c* (Å)	6.7866 (4), 7.1789 (3), 14.0981 (5)
α, β, γ (°)	94.098 (3), 93.461 (4), 106.154 (4)
*V* (Å^3^)	655.75 (5)
*Z*	2
Radiation type	Cu *K*α
μ (mm^−1^)	2.72
Crystal size (mm)	0.12 × 0.12 × 0.08

Data collection	
Diffractometer	Agilent SuperNova Dual Source diffractometer with an Atlas detector
Absorption correction	Multi-scan (*CrysAlis PRO*; Agilent, 2014 ▸)
*T*_min_, *T*_max_	0.737, 0.812
No. of measured, independent and observed [*I* > 2σ(*I*)] reflections	7634, 2409, 2109
*R*_int_	0.022
(sin θ/λ)_max_ (Å^−1^)	0.606

Refinement	
*R*[*F* ^2^ > 2σ(*F* ^2^)], *wR*(*F* ^2^), *S*	0.039, 0.106, 1.05
No. of reflections	2409
No. of parameters	190
H-atom treatment	H-atom parameters constrained
Δρ_max_, Δρ_min_ (e Å^−3^)	0.26, −0.49

Computer programs: *CrysAlis PRO* (Agilent, 2014 ▸), *SHELXS97* (Sheldrick, 2008 ▸), *SHELXL2013* (Sheldrick, 2015 ▸), *PLATON* (Spek, 2009 ▸) and *publCIF* (Westrip, 2010 ▸).

## supplementary crystallographic information

### Crystal data

**Table:**

C_16_H_9_ClO_4_	*Z* = 2
*M**_r_* = 300.68	*F*(000) = 308
Triclinic, *P*1	*D*_x_ = 1.523 Mg m^−^^3^
Hall symbol: -P 1	Melting point: 478 K
*a* = 6.7866 (4) Å	Cu *K*α radiation, λ = 1.54184 Å
*b* = 7.1789 (3) Å	Cell parameters from 3886 reflections
*c* = 14.0981 (5) Å	θ = 6.3–69.1°
α = 94.098 (3)°	µ = 2.72 mm^−^^1^
β = 93.461 (4)°	*T* = 293 K
γ = 106.154 (4)°	Prism, colourless
*V* = 655.75 (5) Å^3^	0.12 × 0.12 × 0.08 mm

### Data collection

**Table:**

Agilent SuperNova Dual Source diffractometer with an Atlas detector	2409 independent reflections
Radiation source: fine-focus sealed tube	2109 reflections with *I* > 2σ(*I*)
Graphite monochromator	*R*_int_ = 0.022
Detector resolution: 5.3048 pixels mm^-1^	θ_max_ = 69.1°, θ_min_ = 6.3°
ω scan	*h* = −8→7
Absorption correction: multi-scan (CrysAlis PRO; Agilent, 2014)	*k* = −8→8
*T*_min_ = 0.737, *T*_max_ = 0.812	*l* = −17→16
7634 measured reflections	

### Refinement

**Table:**

Refinement on *F*^2^	Primary atom site location: structure-invariant direct methods
Least-squares matrix: full	Secondary atom site location: difference Fourier map
*R*[*F*^2^ > 2σ(*F*^2^)] = 0.039	Hydrogen site location: inferred from neighbouring sites
*wR*(*F*^2^) = 0.106	H-atom parameters constrained
*S* = 1.05	*w* = 1/[σ^2^(*F*_o_^2^) + (0.0475*P*)^2^ + 0.1948*P*] where *P* = (*F*_o_^2^ + 2*F*_c_^2^)/3
2409 reflections	(Δ/σ)_max_ < 0.001
190 parameters	Δρ_max_ = 0.26 e Å^−^^3^
0 restraints	Δρ_min_ = −0.49 e Å^−^^3^

### Special details

**Table:**

Geometry. All esds (except the esd in the dihedral angle between two l.s. planes) are estimated using the full covariance matrix. The cell esds are taken into account individually in the estimation of esds in distances, angles and torsion angles; correlations between esds in cell parameters are only used when they are defined by crystal symmetry. An approximate (isotropic) treatment of cell esds is used for estimating esds involving l.s. planes.
Refinement. Refinement of F^2^ against ALL reflections. The weighted R-factor wR and goodness of fit S are based on F^2^, conventional R-factors R are based on F, with F set to zero for negative F^2^. The threshold expression of F^2^ > 2sigma(F^2^) is used only for calculating R-factors(gt) etc. and is not relevant to the choice of reflections for refinement. R-factors based on F^2^ are statistically about twice as large as those based on F, and R- factors based on ALL data will be even larger.

### Fractional atomic coordinates and isotropic or equivalent isotropic displacement parameters (Å^2^)

**Table:**

	*x*	*y*	*z*	*U*_iso_*/*U*_eq_	
C1	0.1899 (3)	0.0796 (2)	0.35706 (12)	0.0446 (4)	
C2	0.3804 (3)	0.0653 (2)	0.31891 (11)	0.0422 (4)	
C3	0.5634 (3)	0.1322 (2)	0.36885 (11)	0.0414 (4)	
H3	0.6815	0.1187	0.3424	0.050*	
C4	0.5771 (2)	0.2250 (2)	0.46354 (11)	0.0384 (3)	
C5	0.7616 (3)	0.3026 (2)	0.52035 (13)	0.0475 (4)	
H5	0.8848	0.2943	0.4975	0.057*	
C6	0.7618 (3)	0.3918 (3)	0.61044 (14)	0.0549 (5)	
H6	0.8856	0.4451	0.6475	0.066*	
C7	0.5794 (3)	0.4022 (3)	0.64594 (12)	0.0534 (5)	
H7	0.5813	0.4628	0.7067	0.064*	
C8	0.3938 (3)	0.3229 (2)	0.59161 (12)	0.0474 (4)	
H8	0.2705	0.3270	0.6158	0.057*	
C9	0.3956 (2)	0.2379 (2)	0.50103 (11)	0.0390 (3)	
C10	0.3047 (3)	0.0312 (3)	0.15135 (12)	0.0459 (4)	
C11	0.2730 (2)	−0.1155 (3)	0.06844 (12)	0.0458 (4)	
C16	0.2973 (3)	−0.2994 (3)	0.07788 (13)	0.0525 (4)	
H16	0.3335	−0.3326	0.1377	0.063*	
C15	0.2683 (3)	−0.4333 (3)	−0.00072 (14)	0.0599 (5)	
H15	0.2832	−0.5568	0.0058	0.072*	
C14	0.2170 (3)	−0.3809 (4)	−0.08894 (14)	0.0626 (6)	
C13	0.1904 (3)	−0.1999 (4)	−0.10068 (13)	0.0649 (6)	
H13	0.1534	−0.1680	−0.1607	0.078*	
C12	0.2200 (3)	−0.0667 (3)	−0.02135 (13)	0.0555 (5)	
H12	0.2042	0.0564	−0.0282	0.067*	
Cl1	0.18104 (10)	−0.55203 (13)	−0.18674 (4)	0.0951 (3)	
O1	0.20802 (17)	0.16486 (17)	0.44814 (8)	0.0444 (3)	
O2	0.0204 (2)	0.0211 (2)	0.31579 (10)	0.0637 (4)	
O3	0.3603 (2)	−0.04346 (18)	0.23221 (8)	0.0516 (3)	
O4	0.2900 (2)	0.1930 (2)	0.15125 (10)	0.0592 (3)	

### Atomic displacement parameters (Å^2^)

**Table:**

	*U*^11^	*U*^22^	*U*^33^	*U*^12^	*U*^13^	*U*^23^
C1	0.0428 (9)	0.0444 (8)	0.0465 (9)	0.0127 (7)	0.0018 (7)	0.0042 (7)
C2	0.0515 (10)	0.0402 (8)	0.0374 (8)	0.0177 (7)	0.0038 (7)	0.0026 (6)
C3	0.0424 (9)	0.0431 (8)	0.0433 (8)	0.0176 (7)	0.0101 (7)	0.0082 (7)
C4	0.0411 (8)	0.0336 (7)	0.0420 (8)	0.0117 (6)	0.0044 (6)	0.0066 (6)
C5	0.0426 (9)	0.0461 (9)	0.0549 (10)	0.0139 (7)	0.0001 (7)	0.0097 (7)
C6	0.0597 (11)	0.0467 (9)	0.0538 (10)	0.0117 (8)	−0.0144 (8)	0.0049 (8)
C7	0.0801 (13)	0.0438 (9)	0.0386 (8)	0.0230 (9)	−0.0015 (8)	0.0018 (7)
C8	0.0601 (11)	0.0458 (9)	0.0420 (8)	0.0230 (8)	0.0084 (7)	0.0057 (7)
C9	0.0417 (8)	0.0346 (7)	0.0428 (8)	0.0134 (6)	0.0045 (6)	0.0069 (6)
C10	0.0369 (9)	0.0587 (10)	0.0442 (9)	0.0156 (7)	0.0051 (7)	0.0082 (7)
C11	0.0341 (8)	0.0648 (11)	0.0388 (8)	0.0140 (7)	0.0044 (6)	0.0048 (7)
C16	0.0515 (10)	0.0651 (11)	0.0407 (9)	0.0181 (8)	0.0000 (7)	0.0006 (8)
C15	0.0541 (11)	0.0709 (12)	0.0519 (10)	0.0169 (9)	0.0015 (8)	−0.0073 (9)
C14	0.0401 (10)	0.0978 (16)	0.0434 (10)	0.0137 (10)	0.0043 (7)	−0.0134 (10)
C13	0.0437 (10)	0.1146 (19)	0.0370 (9)	0.0235 (11)	0.0029 (7)	0.0066 (10)
C12	0.0432 (10)	0.0826 (13)	0.0446 (9)	0.0217 (9)	0.0061 (7)	0.0141 (9)
Cl1	0.0754 (4)	0.1412 (6)	0.0550 (3)	0.0214 (4)	0.0009 (3)	−0.0395 (4)
O1	0.0383 (6)	0.0504 (6)	0.0464 (6)	0.0160 (5)	0.0069 (5)	0.0012 (5)
O2	0.0439 (7)	0.0792 (9)	0.0619 (8)	0.0120 (6)	−0.0056 (6)	−0.0029 (7)
O3	0.0686 (8)	0.0544 (7)	0.0369 (6)	0.0280 (6)	0.0000 (5)	−0.0007 (5)
O4	0.0689 (9)	0.0586 (8)	0.0561 (8)	0.0273 (7)	0.0046 (6)	0.0092 (6)

### Geometric parameters (Å, º)

**Table:**

C1—O2	1.206 (2)	C8—H8	0.9300
C1—O1	1.366 (2)	C9—O1	1.382 (2)
C1—C2	1.456 (2)	C10—O4	1.193 (2)
C2—C3	1.335 (2)	C10—O3	1.370 (2)
C2—O3	1.3809 (19)	C10—C11	1.479 (2)
C3—C4	1.435 (2)	C11—C16	1.390 (3)
C3—H3	0.9300	C11—C12	1.390 (2)
C4—C9	1.393 (2)	C16—C15	1.381 (3)
C4—C5	1.395 (2)	C16—H16	0.9300
C5—C6	1.380 (3)	C15—C14	1.377 (3)
C5—H5	0.9300	C15—H15	0.9300
C6—C7	1.382 (3)	C14—C13	1.381 (4)
C6—H6	0.9300	C14—Cl1	1.738 (2)
C7—C8	1.385 (3)	C13—C12	1.385 (3)
C7—H7	0.9300	C13—H13	0.9300
C8—C9	1.378 (2)	C12—H12	0.9300
			
O2—C1—O1	118.19 (16)	C8—C9—C4	122.08 (16)
O2—C1—C2	125.73 (17)	O1—C9—C4	120.91 (14)
O1—C1—C2	116.07 (14)	O4—C10—O3	122.83 (17)
C3—C2—O3	120.59 (15)	O4—C10—C11	127.29 (16)
C3—C2—C1	122.67 (15)	O3—C10—C11	109.86 (15)
O3—C2—C1	116.26 (15)	C16—C11—C12	119.31 (18)
C2—C3—C4	119.71 (15)	C16—C11—C10	121.73 (16)
C2—C3—H3	120.1	C12—C11—C10	118.96 (18)
C4—C3—H3	120.1	C15—C16—C11	120.64 (18)
C9—C4—C5	118.11 (15)	C15—C16—H16	119.7
C9—C4—C3	118.07 (15)	C11—C16—H16	119.7
C5—C4—C3	123.81 (15)	C14—C15—C16	118.9 (2)
C6—C5—C4	120.22 (17)	C14—C15—H15	120.5
C6—C5—H5	119.9	C16—C15—H15	120.5
C4—C5—H5	119.9	C15—C14—C13	121.89 (19)
C5—C6—C7	120.47 (17)	C15—C14—Cl1	118.0 (2)
C5—C6—H6	119.8	C13—C14—Cl1	120.05 (16)
C7—C6—H6	119.8	C14—C13—C12	118.72 (18)
C6—C7—C8	120.40 (17)	C14—C13—H13	120.6
C6—C7—H7	119.8	C12—C13—H13	120.6
C8—C7—H7	119.8	C13—C12—C11	120.5 (2)
C9—C8—C7	118.70 (17)	C13—C12—H12	119.7
C9—C8—H8	120.6	C11—C12—H12	119.7
C7—C8—H8	120.6	C1—O1—C9	122.54 (13)
C8—C9—O1	117.01 (15)	C10—O3—C2	118.78 (14)
			
O2—C1—C2—C3	−179.51 (17)	O4—C10—C11—C12	0.0 (3)
O1—C1—C2—C3	−0.5 (2)	O3—C10—C11—C12	−178.60 (15)
O2—C1—C2—O3	−7.4 (3)	C12—C11—C16—C15	−0.3 (3)
O1—C1—C2—O3	171.59 (13)	C10—C11—C16—C15	−179.53 (16)
O3—C2—C3—C4	−172.69 (13)	C11—C16—C15—C14	0.7 (3)
C1—C2—C3—C4	−0.9 (2)	C16—C15—C14—C13	−1.1 (3)
C2—C3—C4—C9	1.5 (2)	C16—C15—C14—Cl1	−179.94 (15)
C2—C3—C4—C5	−178.72 (15)	C15—C14—C13—C12	1.1 (3)
C9—C4—C5—C6	−1.0 (2)	Cl1—C14—C13—C12	179.94 (14)
C3—C4—C5—C6	179.23 (15)	C14—C13—C12—C11	−0.7 (3)
C4—C5—C6—C7	1.1 (3)	C16—C11—C12—C13	0.3 (3)
C5—C6—C7—C8	0.2 (3)	C10—C11—C12—C13	179.57 (16)
C6—C7—C8—C9	−1.5 (3)	O2—C1—O1—C9	−179.48 (15)
C7—C8—C9—O1	−178.39 (14)	C2—C1—O1—C9	1.4 (2)
C7—C8—C9—C4	1.6 (2)	C8—C9—O1—C1	179.09 (14)
C5—C4—C9—C8	−0.4 (2)	C4—C9—O1—C1	−0.9 (2)
C3—C4—C9—C8	179.42 (14)	O4—C10—O3—C2	6.6 (3)
C5—C4—C9—O1	179.60 (13)	C11—C10—O3—C2	−174.68 (14)
C3—C4—C9—O1	−0.6 (2)	C3—C2—O3—C10	−115.15 (18)
O4—C10—C11—C16	179.25 (18)	C1—C2—O3—C10	72.60 (19)
O3—C10—C11—C16	0.6 (2)		
